# Effects of resistance training on the glycemic control of people with type 1 diabetes: a systematic review and meta-analysis

**DOI:** 10.20945/2359-3997000000487

**Published:** 2022-06-27

**Authors:** Valderi de Abreu de Lima, Francisco José de Menezes, Luana da Rocha Celli, Suzana Nesi França, Gabriel Ribeiro Cordeiro, Luis Paulo Gomes Mascarenhas, Neiva Leite

**Affiliations:** 1 Universidade Federal do Paraná Departamento de Educação Física Núcleo de Qualidade de Vida Curitiba PR Brazil Universidade Federal do Paraná (UFPR), Departamento de Educação Física, Núcleo de Qualidade de Vida, Curitiba, PR, Brasil; 2 Universidade Federal do Paraná Departamento de Pediatria Unidade de Endocrinologia Pediátrica Curitiba PR Brazil Universidade Federal do Paraná (UFPR), Departamento de Pediatria, Unidade de Endocrinologia Pediátrica, Curitiba, PR, Brasil; 3 Universidade Estadual do Centro--Oeste Departamento de Educação Física Programa de Pós-graduação Interdisciplinar em Desenvolvimento Comunitário Irati PR Brazil Universidade Estadual do Centro--Oeste (Unicentro), Departamento de Educação Física, Programa de Pós-graduação Interdisciplinar em Desenvolvimento Comunitário, Irati, PR, Brasil

**Keywords:** Chronic disease, metabolism, physical activity

## Abstract

Resistance training has shown the potential to contribute to better glycemic control in people with Type 1 Diabetes (T1D), however, there are contradictory results in this regard and a need to clarify the effects of isolated resistance training on glycemic control in T1D. The aim was to verify the effects of resistance training on the glycemic control of people with T1D. Original articles were selected, randomized and non-randomized clinical trials that aimed to verify chronic responses, through the concentrations of glycated hemoglobin (HbA1c), to a structured program of resistance exercise in the glycemia of patients with T1D. The following databases were searched; MEDLINE, PubMed, Web of Science, Scopus, ScienceDirect, LILACS, and SciELO. Five studies were included in the review. A reduction in HbA1c was observed (SMD = -0.568 ± 0.165 [95% CI = -0.891 to -0.246]; p = 0.001; I² = 82%) in patients undergoing resistance training, when compared to the control group (SMD = 1.006 ± 0.181 [95% CI = 0.653 to 1.360]; p <0.001). Two studies, with children and adolescents and longer interventions, demonstrated a significant reduction in HbA1c, increased strength, and an improved lipid profile. Resistance training was efficient for assisting in glycemic control in people with T1D and should be incorporated in treatment plans.

## INTRODUCTION

Diabetes mellitus is a chronic disease in which there are disturbances in carbohydrate metabolism, due to deficiency in pancreatic insulin production and secretion, in type 1 diabetes (T1D), or its peripheral action, type 2 diabetes. In both forms, the disease is characterized by chronic hyperglycemia and changes in the metabolism of carbohydrates, lipids, and proteins in relation to the action and presence of insulin ( 1 , 2 ).

To assist in the glycemic control of people with T1D, the association of a healthy lifestyle with a balanced diet and regular physical activity is recommended, in addition to the administration of exogenous insulin and regular monitoring of blood glucose ( 3 ). Thus, physical exercise, defined as structured and planned body movements, is used as a management and treatment strategy for T1D ( 4 , 5 ). During exercise, muscle contractions can increase the permeability of the cell membrane, with an insulin-like effect, leading to greater sensitivity to exogenous insulin ( 6 ).

The treatment of T1D, in addition to the use of exogenous insulin, involves dietary care and the prescription of physical exercises, especially aerobic exercise ( 7 ). However, resistance exercise can help maintain and increase muscle mass and has been proposed to assist in glycemic control, since the muscle is the optimal area of use of surplus energy substrates and insulin. In addition, resistance exercise causes less glucose decline during activity and is associated with longer-lasting reductions in post-exercise blood glucose than aerobic exercise ( 4 , 8 ). With the increase in muscle mass, there is greater glucose uptake, a reduction in hyperglycemias, and less need for exogenous insulin ( 9 ). Therefore, it is important to clarify the effects of isolated resistance exercises on glycemic control in T1D.

Control of the hyperglycemia caused by the disease is extremely important, because when poorly managed, in the long term, it generates macrovascular complications (ischemic heart disease, peripheral arterial disease, and stroke) and microvascular complications (retinopathy, neuropathy, and nephropathy), which affect the quality of life of people with diabetes and cause individual and collective costs for health systems ( 10 , 11 ). In this context, glycated hemoglobin (HbA1c) tests are often used to assess glycemic control.

HbA1c is considered the gold standard test to assess the glycemic control of individuals with T1D, since the relationship between increased glucose levels and risk of microvascular complications has been consistently demonstrated. The determination of HbA1c makes it possible to estimate how high blood glucose levels were in the previous 3 to 4 months ( 12 ). Studies are controversial on the effect of resistance training on HbA1c levels in patients with T1D ( 13 , 14 ).

The majority of studies analyze the effects of aerobic exercise and show favorable results on the acute management of several clinical outcomes. However, resistance exercise presents adversarial responses regarding the effect on the metabolic control of HbA1c and other clinical variables, and has been less widely researched ( 4 , 13 ). in this way, there is a lack of information about isolated resistance exercise in controlling glycemia in people with T1D and a meta-analysis could improve the statistical power of research on the effects of treatments and increase the accuracy of estimated effect sizes of isolated resistance exercise in glycemic control of T1D. Based on this, the present study aimed to conduct a systematic review with meta-analysis to verify the effects of isolated resistance training on the glycemic control of people with T1D.

## METHODS

The current research is a systematic review of the literature with meta-analysis that seeks to analyze the effects of resistance exercise prescribed in isolation in the control T1D. This study is registered on PROSPERO under reference number CRD42020170439.

The following descriptors were used, indexed by Medical Subject Headings (MeSH): “Type 1 Diabetes” OR “type 1 diabetics” AND “exercise” OR “resistance training” OR “strength training” OR “weight training” on the platforms MEDLINE (Medical Literature Analysis and Retrieval System online), PubMed, Web of Science, Scopus, ScienceDirect, LILACS (Latin American and Caribbean Center for Science Information of Health), and SciELO.

The titles and abstracts of all articles published in the last fifteen years were analyzed and those that met the inclusion criteria were selected. If there was an insufficient amount of information provided by the summary, the study was read in full. Furthermore, the reference lists of the studies was searched for additional studies in agreement with the criteria.

Original studies, randomized and non-randomized clinical trials were included, that aimed to verify chronic glycemic responses to a structured resistance exercise program in patients with T1D.

Regarding the exclusion criteria, review studies, studies with animals, case studies, letters to the editor, abstracts published in congresses, studies that used other types of exercises or did not show results of glycemic control, assessed by HbA1c, after the intervention, were excluded.

### Quality analysis of the articles

The methodological quality of the studies was assessed using an instrument adapted from the PEDro scale for studies with randomized clinical trials. The protocol consisted of 8 criteria, as shown in Table 1 . For each criterion, a value of 1 or 0 was assigned when the criterion was met, or not, respectively. According to the total score, studies with the scores: <5, 6, and >7 were classified as high, moderate, and low risk of bias, respectively.

**Table 1:** Methodological quality of the selected studies

Author/criterion	1	2	3	4	5	6	7	8	Total
Wróbel et al., 2018	1	1	0	1	1	1	1	1	7
Petschnig et al., 2020	1	1	1	1	1	1	1	1	8
Salem et al., 2010	1	1	1	1	1	1	1	1	8
Toghi-Eshghi and Yardley, 2019	1	1	0	1	1	1	1	1	7
Ramalho et al., 2006	1	1	0	1	1	1	1	1	7

Note: Criteria: 1. Eligibility criteria were specified; 2. Subjects were randomly assigned to groups; 3. There was a control group; 4. Initially, the groups were similar with respect to the most important prognostic indicators; 5. The sample assigned to groups according to characteristics was homogeneous; 6. The intervention methodology is clearly described; 7. The results of inter-group statistical comparisons were described for at least one key outcome; 8. All subjects from which outcome measures were presented received the treatment or control condition as allocated or, when this was not the case, data analysis was performed for at least one of the key outcomes by “intention-to-treat”.

SOURCE: The author (2021).

### Statistical analysis

The groups of each study, with and without interventions (control, no exercise) were compared, the main outcome being the control of glycemia. The measures of effect used are the mean and standard deviation assessed before and after the intervention. The information collected from the study was divided between sample characteristics (total participants, age, and sex), resistance training protocol (frequency, volume, exercise, and intensity) and glycemic control (HbA1c). The data were analyzed by two researchers independently.

For the meta-analysis, the software Comprehensive Meta-Analysis (v.2.2.064) was used. The effect size of the interventions was obtained by the difference between the pre- and post-intervention period for intervention studies and baseline values in comparative studies. Subsequently, the standardized mean difference (SMD) and standard error (SE) were calculated and adjusted for all groups. The significance level was set at p < 0.5 and a 95% confidence interval (CI) was considered. In addition, the analysis of heterogeneity between the studies was obtained by the I² test, in which I² < 25%, 25%-50%, and > 50% were considered as small, medium, and large inconsistencies, respectively ( 15 ). The publication bias was conducted based on the funnel plot and the Egger test ( 16 ). Sensitivity analysis was performed by removing a study from the analysis.

## RESULTS

**Figure 1:**
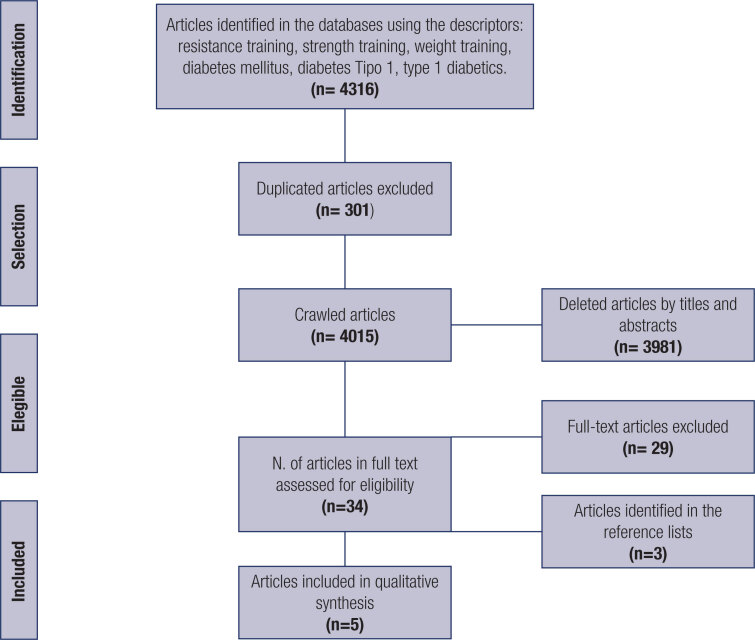
Prisma flow diagram.

Source: The author (2021).

Initially 4316 studies were identified in the database searches, of which 301 duplicates were excluded, leaving 4015 for analysis. After reading the titles, 380 remained for the abstract reading, of which 34 were selected, resulting in 3,981 excluded articles. The selected studies were read in full, leading to 29 exclusions. A further 3 studies were included after being identified in the reference lists, and finally, 5 articles met the inclusion criteria and did not meet any exclusion criteria, thus being eligible for the review ( Figure 1 ).

Considering the characteristics of the articles eligible for the research, a tendency towards small samples was noted, except for one study, which included 75 participants. The mean age and periodization of each study varied, and the duration of the interventions was between 12 and 32 weeks ( Table 2 ).

**Table 2:** Characteristics of the studies, including sample, training, intervention time (weeks), and main results

Authors	Sample (n)	Age	Periodization	Weeks	Main results
Wróbel et al., 2018	11	35 ± 6	5 exercises 5 sets, 10 to 15 repetitions, 50% 1 MR (2 min interval)	12	Tendency to increase in VO_2max_, without altering the HR_max_, suggests benefit to cardiorespiratory system. Downward trend in HbA1c with no statistical significance.
Petschnig et al., 2020	11	11 ± 0.8	Start with 30% 1 MR, intensity changed every 15 days. Circuit of 20-40 min of training, 8 stations, 180 s of rest. Each exercise lasted 25-40 s, with 40 to 30 s of rest.	32	Increased strength of upper and lower limbs. Significant reduction in HbA1c after 32 weeks. Reduction in blood glucose after training sessions and increase in adiponectin.
Salem et al., 2010	75	14.7 ± 2.2	1 x 10 repetitions x 50%load of 10 MR + 10 repetitions x 75% load of 10 MR + 10 repetitions x 100% load of 10 MR (2 min break)	24	Significant improvement in HbA1c. Decreased insulin requirement, reduced BMI and waist circumference. Elevated concentrations of HbA1c associated with increased LDL, cholesterol, and TGD. No change in hypoglycemic episodes, or BP, only in diastolic BP.
Toghi-Eshghi and Yardley, 2019	12	31.3 ± 8.9	7 exercises, 3 sets, 8 repetitions, 100 % 8 MR (90 s of rest)	12	Increased blood glucose with exercise on an empty stomach and decreased in the afternoon. After 60 minutes, fasting blood glucose was significantly higher than in the afternoon. Greater variability in blood glucose after exercise performed on an empty stomach. Tendency to increase in HbA1c.
Ramalho et al., 2006	6	19.8 ± 5.1	8 exercises, 3 sets, 8-12 repetitions, 60-80% 1 MR (60 s break)	12	There was no change in the parameters evaluated, only a reduction in insulin dosage after exercise.

Source: The authors (2021).

The methodological quality of the articles ( Table 1 ) was evaluated using an evaluation protocol designed for this study, adapted from the PEDro Scale ( 17 ).

**Figure 2:**
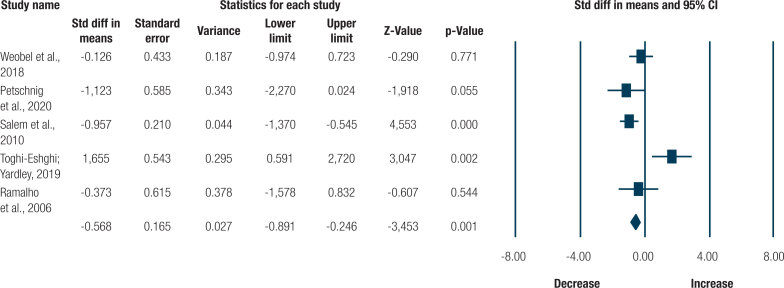
Effect size analysis for the resistance training group

Source: The author (2021).

**Figure 3:**
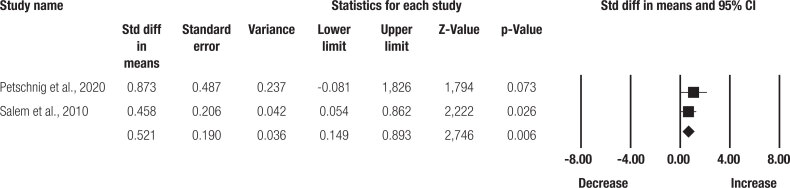
Effect size analysis for the control group.

Source: The author (2021).

Five studies verified the effect of resistance training programs on HbA1c ( 16 , 17 , 18 , 19 ). The data of 115 participants were analyzed. According to the meta-analysis, a relative risk for a reduction in HbA1c was observed in the resistance training group (SMD = -0.568 ± 0.165 [95%CI = -0.891 to -0.246]; p = 0.001; I² = 82%; Figure 2 ) and an increase for the control group (SMD = 0.521 ± 0.190 [95%CI = 0.149 to 0.893]; p = 0.006; I² = 00%; Figure 3 ).

**Figure 4:**
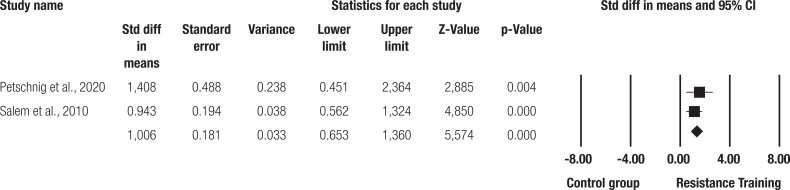
Comparison of effect size between the resistance training group and the control group.

Source: The author (2021).

In addition, when comparing the groups, a significant relative risk of reduction in HbA1c concentrations was observed in favor of the resistance training group compared to the control group (SMD = 1.006 ± 0.181 [95%CI = 0.653 to 1.360]; p < 0.001; Figure 4 ), with small inconsistencies between the results (I² = 00%, p = 0.376).

No significant publication bias was detected in the asymmetry of the funnel graph or in the Egger test (p = 0.308). According to the sensitivity analysis for the resistance training group ( Figure 3 ), heterogeneity reduced significantly between the groups to 20% (p = 0.287), after removing one study ( 18 ). Due to the reduced number of studies for the other analyses, it was not possible to conduct a sensitivity analysis.

## DISCUSSION

The purpose of this systematic review was to analyze the effect of resistance training on the metabolic control of people with T1D. The meta-analysis showed better control of HbA1c in people submitted to resistance training, as well as in other clinical variables.

In this regard, controversial results are found in the literature, some studies observed an improvement in glycemic control in individuals with T1D, while others did not. Durak and cols. ( 19 ), reported improvement in HbA1c after a resistance exercise program, corroborating with what was found in the current research. The majority of studies, however, analyzed the combined effect of aerobic and resistance exercise and reported contradictory results ( 20 - 22 ).

Among the five studies analyzed in this review, Wróbel and cols. ( 23 ) and Ramalho and cols. ( 13 ) did not find a significant reduction in HbA1c, but the authors did observe a downward trend. This can be explained by the intervention time (both of 12 weeks), since shorter interventions suggest less effectiveness. The studies by Salem and cols. ( 24 ) and Petschnig and cols. ( 14 ), with 24 and 32 weeks of interventions respectively, found significant improvements in this parameter. In addition, the age group may have influenced the results, as the first two studies worked with the adult population, the latter two were aimed at children and adolescents.

It is known that growth and maturation have an influence on insulin sensitivity, mainly due to morphological aspects ( 25 ). Thus, the increase in muscle strength, as reported by Petschnig and cols. ( 14 ), may have optimized anaerobic metabolism during exercise, using a greater amount of glucose in these patients when compared to the control group, which would explain the significant reduction in HbA1c after the intervention.

Maintenance of whole-body glucose homeostasis is the result of a complex regulatory system involving various tissues, of which skeletal muscle is the main site of glucose uptake, release, and storage ( 26 ). This occurs because it is also related to the better use of surplus substrates due to the action of insulin ( 8 ). Thus, the increase in muscle mass may be related to better metabolic glycemic control, which could also explain why longer training sessions have a greater effect on HbA1c. The results of Petschnig and cols. ( 14 ), supported this hypothesis, reporting a significant increase in upper and lower limb strength and a significant reduction in HbA1c after 32 weeks of training.

It should also be noted that the mechanisms of glucose uptake in skeletal muscle are enhanced for up to 48 hours after training ( 12 ), therefore increasing insulin sensitivity and decreasing the need for its exogenous administration. Thus, the results found by Salem and cols. ( 24 ) and Ramalho and cols. ( 13 ), of decreased insulin doses after training also demonstrate better long-term metabolic control of HbA1c.

There was inconsistency in the study by Toghi-Eshghi and Yardley ( 18 ), while all the others identified a reduction in the effect size analysis, these authors reported an increase. One hypothesis for this occurrence is that, in this case, the patients performed the training protocol at two different moments: fasting at 7am and fed during the afternoon. Fasting exercise favored an increase in blood glucose, which may have altered the long-term results. The authors argue that this increase in blood glucose may be related to natural changes in blood glucose in the morning, and differences in circulating insulin due to administration or different insulin sensitivities throughout the day, but point out that the small sample did not allow precise conclusions to be obtained.

The hypothesis of Petschnig and cols. ( 14 ) that there would be an improvement in adiponectin concentrations was confirmed by the study. The authors explain that adiponectin is related to insulin sensitivity, and thus, increased blood concentrations are beneficial to individuals with diabetes.

Another positive point observed is related to hypoglycemia. Hypoglycemia is still one of the main barriers to exercise in people with T1D ( 27 ). Resistance exercise suggested less risk of hypoglycemic episodes in the study by Jimenez and cols. ( 28 ), and in that case, insulin sensitivity remained unchanged for 36 hours after exercise. The study by Salem and cols. ( 24 ), confirms this result, as there was no change in episodes of hypoglycemia related to training.

Finally, Salem and cols. ( 24 ), demonstrated that high concentrations of HbA1c are related to higher LDL, total cholesterol and TGD, demonstrating the importance of controlling this variable. Poor control, on the other hand, is associated with the development of acute and chronic complications of T1D ( 20 ).

From this analysis, it can be considered that the recommendations of Farinha and cols. ( 29 ), that resistance exercise or high intensity aerobic exercise are the best alternatives for HbA1c control in patients with T1D, are supported and should be further explored.

Results on the effect of resistance training in people with T1D are still scarce in the literature, and most studies include a small sample or analyze the acute effects on blood glucose. However, it is known that the best parameter for the analysis of metabolic control is HbA1c ( 12 ).

In addition, it should be noted that there is a great influence of food on the HbA1c result ( 12 ), as well as difficulty controlling this variable in studies investigating the effect of exercise on HbA1c, which could be considered a limiting factor in this area of research. Of the studies presented, only two used some form of control over food ( 18 , 23 ), and both studies showed a tendency to reduced glucose, without a significant difference. In addition, none of the included articles offered nutritional guidelines. Therefore, it is suggested that the participants maintain their usual eating habits during the intervention.

Based on the above, future works should include investigations on the effects of resistance training, paying more attention to greater control of methodological variables and blood glucose parameters, especially HbA1c. Furthermore, larger samples are needed, with nutritional guidance and comparisons with control groups and other types of exercise, which should contribute to better elucidating the relationship between glycemic control and resistance training.

Meta-analysis can improve the statistical power of research on the effects of treatments, being more accurate in estimating the effect size. Furthermore, in cases of apparently discordant results, a meta-analysis allows the attainment of an overview of the situation. In this way, the present meta-analysis demonstrated positive results in relation to glycemic control after resistance training. This may be associated with increased sensitivity to insulin and muscle mass; however, the studies do not present sufficient data on body composition and sensitivity to insulin to verify this hypothesis.

In conclusion, this meta-analysis review identified an improvement in glycemic control, with a reduction in HbA1c concentrations in individuals undergoing resistance training. Thus, it is concluded that resistance training could be an alternative to assist in glycemic control in people with T1D and should be incorporated in treatment plans.
